# Postoperative Hypoparathyroidism in Thyroid Surgery: Anatomic-Surgical Mapping of the Parathyroids and Implications for Thyroid Surgery

**DOI:** 10.1038/s41598-019-52189-3

**Published:** 2019-10-30

**Authors:** Florian Burger, Helga Fritsch, Marit Zwierzina, Rupert Prommegger, Marko Konschake

**Affiliations:** 1 Institute of Clinical and Functional Anatomy, Department for Anatomy, Histology and Embryology, Medical University of Innsbruck (MUI), Innsbruck, Austria; 2 Department of Plastic, Reconstructive and Aesthetic Surgery, Center of Operative Medicine, Medical University of Innsbruck (MUI), Innsbruck, Austria; 3 General and Endocrine Surgery, Sanatorium Kettenbrücke der Barmherzigen Schwestern GmbH, Innsbruck, Austria

**Keywords:** Anatomy, Parathyroid glands

## Abstract

Hypoparathyroidism remains one of the most common complications in thyroid surgery. This study aims for an improved understanding of the complexity of the blood supply and the localisation of the parathyroids compared to the two most important intraoperative landmarks: the inferior laryngeal nerve (ILN) and Zuckerkandl’s tubercle (ZT). We examined 103 laryngeal compounds to classify the blood supply and the localisation of the parathyroids. For intraoperative localisation we defined a Cartesian coordinate system with the ZT plane as x-axis and the course of the inferior laryngeal nerve as y-axis. The inferior thyroid artery (ITA) mainly supplies the parathyroids, whereas the superior thyroid artery provides a backup supply. It must be pointed out that 8.2% of parathyroids receive their blood directly from the thyroid gland. 73.5% of all parathyroids lie within 1 cm of the ILN and 1 cm cranial and 2.5 cm caudal to the ZT plane. Our described perimeters mark the most crucial areas during surgery and provide the surgeon with an anatomic mapping showing areas of special carefulness needed. One should keep bearing in mind all possible blood supply types of the parathyroids and therefore all branches should be handled with care.

## Introduction

Transient or even permanent hypoparathyroidism next to recurrent and/or inferior laryngeal nerve (ILN) palsy remain the most common and important complications in thyroid surgery^1^. The strategies preserving the parathyroids improved over the years: Magnifying glasses, modern sealing instruments and adapted techniques enhanced the postoperative outcome, but the evaluation and decision for an autografting of suspicious parathyroids underlie the experience of the surgeon. Studies about the assessment of the intraoperative change of colour and the accompanying deduction of condition of the parathyroids showed different results^2,3^. A promising approach might be the intraoperative measure of PTH, due to the fact that the level of the hormone during surgery possesses a high predictive value whether a postoperative hypocalcaemia will occur or not^4–6^. Further, the intraoperative use of indocyanine green, in order to evaluate the vascularisation, showed hard correlation between the condition of blood supply and postoperative outcome^7,8^. Furthermore it is difficult for the surgeon to differentiate between parathyroids and other structures intraoperativley, such as lymph nodes or thymic glands^7,8^. This outlines the importance of anatomic knowledge of this region.

Already 110 years ago, Halsted *et al*. and in 1982 Delattre *et al*. described the blood supply of the parathyroids, demonstrating, that the main blood supply is provided by the inferior thyroid artery (ITA), often also having anastomoses from the superior thyroid artery (STA)^9,10^. Nobori *et al*. argue that connections between both the ITA and STA exist nearly always and in about 45% these branches are quite distinct^11^. Other publications describe relevant anastomoses in 20% of the cases^12–15^. During our literature research, a lack of anatomic and surgical studies about the exact blood supply of the parathyroids in combination with a surgical mapping for a surgical application was missing. Both, the blood supply and the localisation of the parathyroids are crucial factors for preservation during surgery, but anatomic variability in this area is high, demanding great experience and flexibility from the surgeon.

Therefore, the aim of this study was to develop a better understanding of the complexity and topography of the blood supply of the parathyroids and their most common variations as well as of the localisation of the parathyroids compared to the two most important intraoperative landmarks: the ILN and the Zuckerkandl tubercle (ZT)^16,17^. This mapping may help the surgeon intraoperatively creating possible intraoperative strategies and algorithms and preventing future patients of a possible transient or even permanent postoperative hypoparathyroidism.

## Results

**Figure 1:**
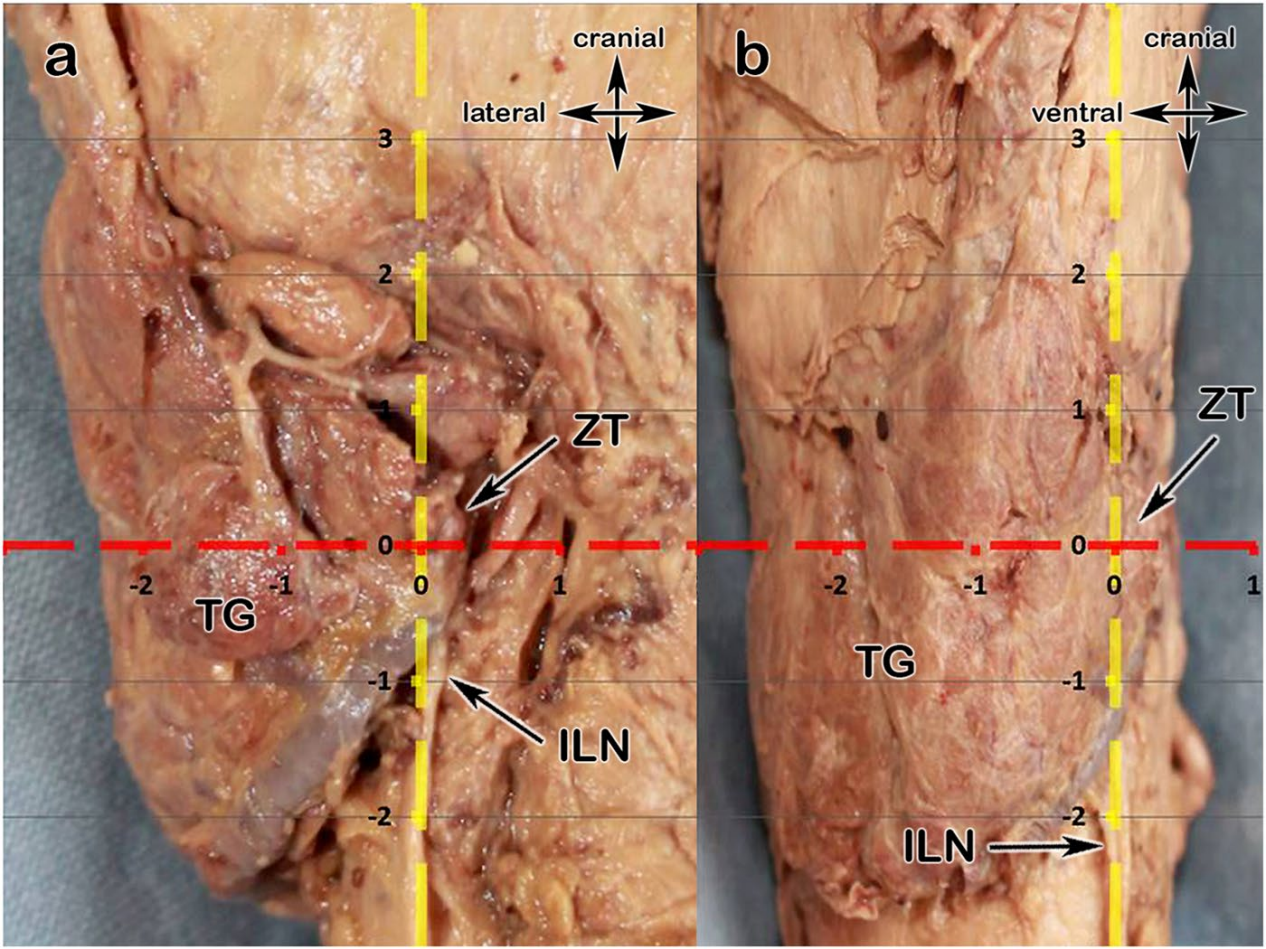
(**a**,**b)** Showing the anatomical mapping (Cartesian coordinate system); **1a** View from dorsal onto the left TG, **2b** view from lateral onto the left TG; thyroid gland (TG), level of the Zuckerkandl tubercle (ZT) – x-axis, inferior laryngeal nerve (ILN) – y-axis.

**Figure 2:**
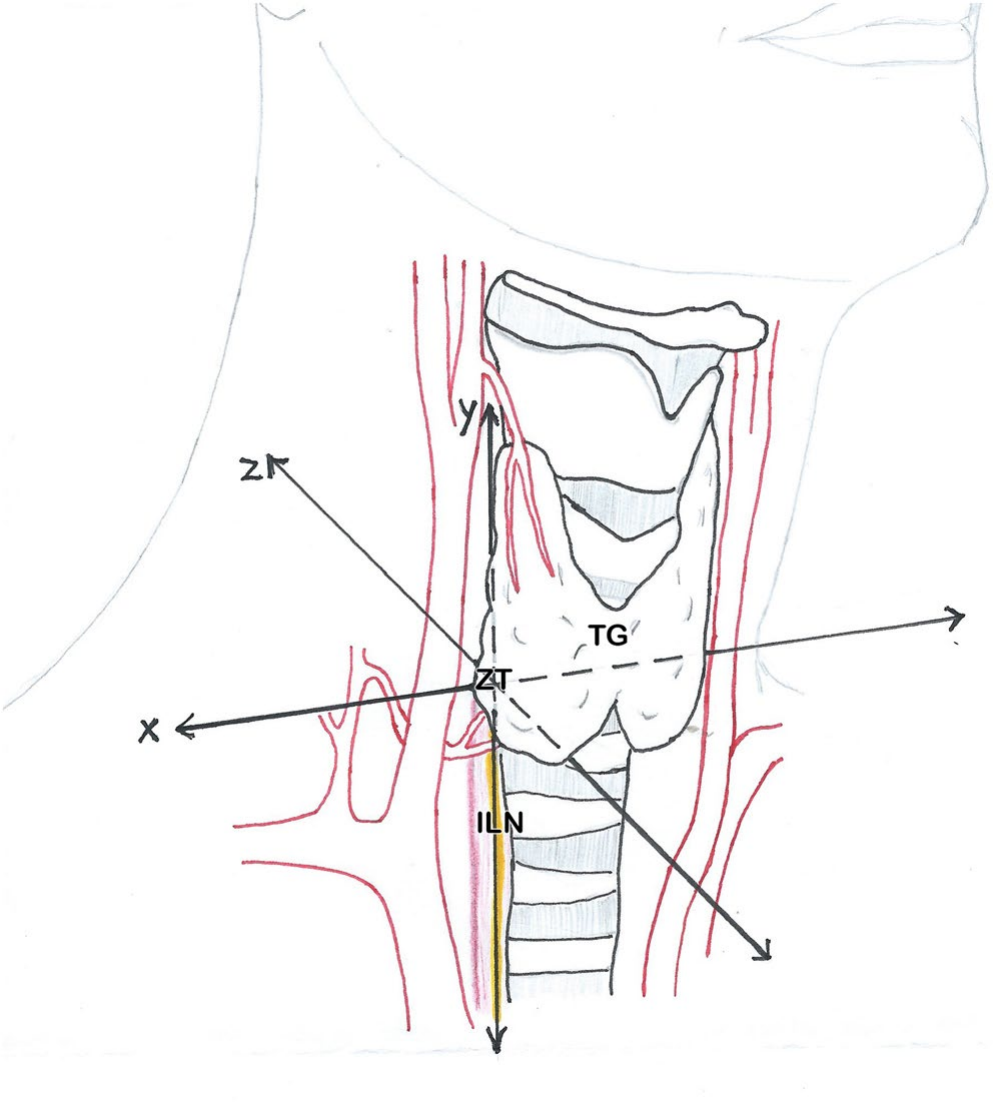
Scetch of the surgical situs; x - medial-lateral axis; y - cranial-caudal axis; z - anterior-posterior axis, ZT - Zuckerkandl tubercle, ILN - inferior laryngeal nerve (yellow).

The two most important intraoperative landmarks, the ILN and the ZT, were shown as an anatomic mapping in Fig. 1a,b; we also provide a scetch for a better overview in Fig. 2.

We have found 317 parathyroids which mean an average of 3.08 parathyroids per laryngeal compound. There have been 32 dissections with four parathyroids, 27 with three, 22 with two and 14 with one. Eight compounds had more than four parathyroids. The fact that we didn’t look for ectopic or intrathyroidal parathyroids may explain this wide variation.

**Figure 3:**
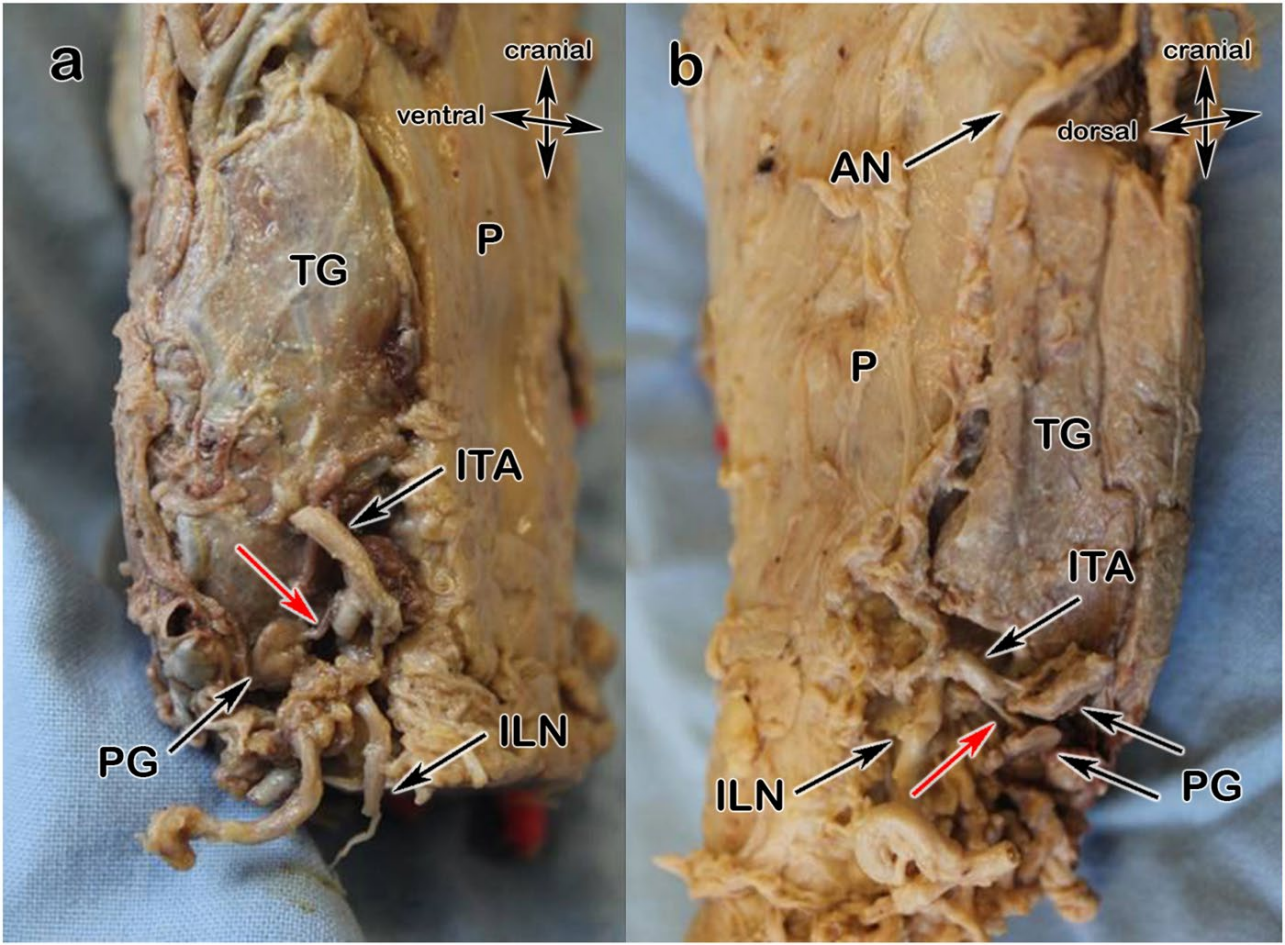
(**a**,**b**) 3a Type a, 3b Type b; pharynx (P), thyroid gland (TG), inferior laryngeal nerve (ILN), inferior thyroid artery (ITA), superior thyroid artery (STA), anastomose (AN); parathyroids (PG), single arrows show supplying arteries.

**Figure 4:**
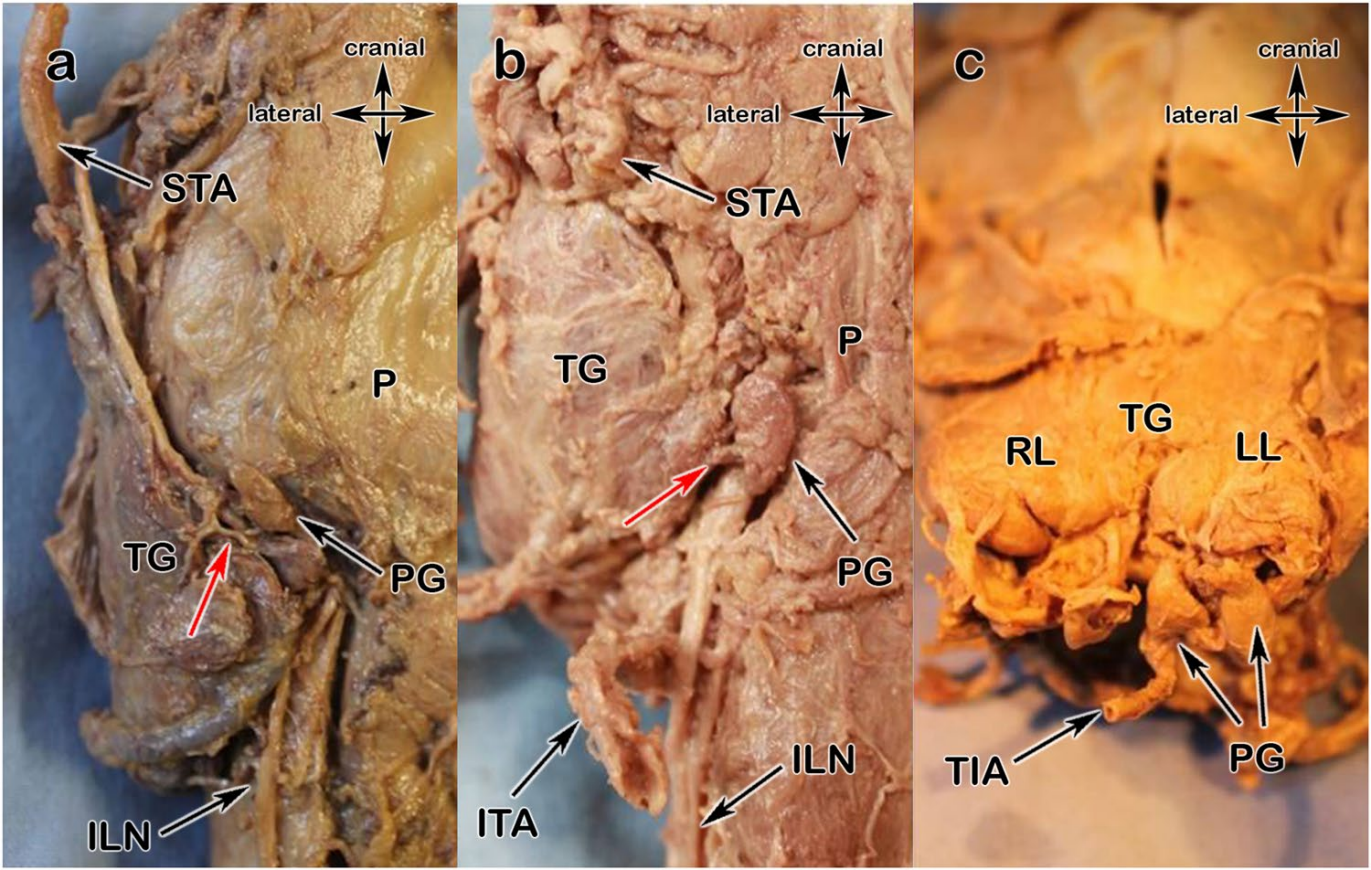
(**a**–**c)** 4a Type x: blood supply by the STA, **4b** blood supply directly out of the TG, **4c** blood supply by the TIA; right thyroid lobe (RL), left thyroid lobe (LL), thyroid ima artery (TIA), inferior laryngeal nerve (ILN), parathyroids (PG), single arrows show supplying arteries.

According to the evaluated blood supply we suggest defining 3 types, type a, type b and type x:

Type a (Fig. 3a) is the most common type: the parathyroids receive their blood from the inferior thyroid artery (ITA). Very small subcapsular or intrathyroidal anastomoses have not been counted due to the fact, that they cannot be preserved during thyroidectomy. Type b (Fig. 3b) describes the blood supply from the ITA with anastomoses from the STA. The anastomoses have only been recorded when epi- or intracapsular vessels were thick enough for being observed without magnifying glasses, so that a sufficient blood supply is ensured in the case of the ligation of a delivering vessel. Type x (Fig. 4a–c), with its three subtypes, is characterized by exclusive supply from another vessel, so as the STA, directly from the thyroid gland (TG) or from the thyroid ima artery (TIA).

### Superior parathyroid glands

The left and right superior parathyroids are mainly supplied by the ITA (40.2% and 65.8%) but there were anastomoses in 26% and 23.7% of the cases with the STA. In 33.8% and 10.5% the superior parathyroids received their blood from another vessel. Nearly 18.2% and 2.6% are exclusively supplied by the STA. It must be pointed out, that 14.3% and 8% received blood exclusively from the thyroid gland. In one case we found a supply by a TIA. So, the superior parathyroids were supplied in 52.9% by the ITA, anastomoses with the STA existed in 24.9%. 22.2% of the superior parathyroids were type x; 11.1% received their blood directly from the thyroid gland (Table 1). No symmetry of the blood supply between left and right was observed; 39% of the superior parathyroid glands received blood out of the same vessels on both sides, 61% didn’t.

**Table 1:** Types of blood supply.

	Amount	Type a	Type b	Type x	*TG*	*STA*	*TIA*
left SPG	77	40.2%	26%	33.8%	*14*.*3%*	*18*.*2%*	*1*.*3%*
left IPG	76	72.4%	7.9%	19.7%	*4%*	*11*.*8%*	*3*.*9%*
right SPG	76	65.8%	23.7%	10.5%	*7*.*9%*	*2*.*6%*	—
right IPG	88	82.9%	5.7%	11.4%	*6*.*8%*	*2*.*3%*	*2*.*3%*

### Inferior parathyroid glands

The left and right inferior parathyroid (IPG) were mainly supplied by the ITA (72.4% and 82.9%) but there were anastomoses in about 7.9% and 5.7% of the cases with the STA. 19.7% and 11.4% of the inferior parathyroids received blood from the thyroid gland (4% and 6.8%) or were exclusively supplied by the STA (11.8% and 2.3%). In two cases we found supply by a TIA. This means a blood supply in 78% from the ITA, with anastomoses in 6.8% of the cases. We found type x in 15.2% with an exclusive supply from the thyroid gland in 5.5% (Table 1). No significant symmetry of the blood supply between left and right has been observed; 66% of the superior parathyroid glands received blood out of the same vessels on both sides, 34% didn’t.

**Figure 5:**
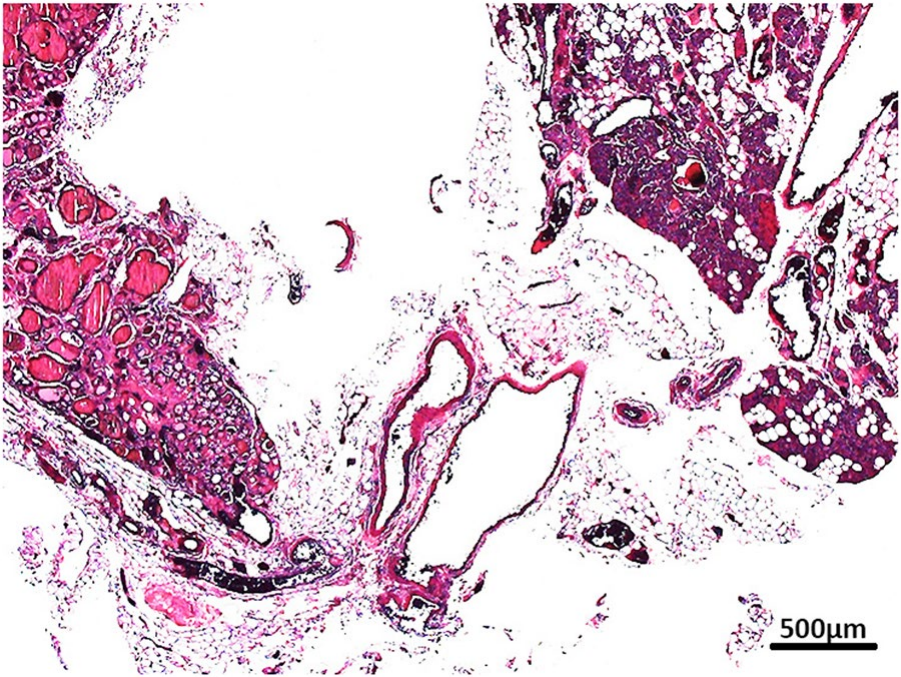
Histological prove of parathyroid tissue and supplying vessels.

Overall, the ITA is the main supply artery with 65.9%, the STA being involved via anastomotic vessels in 15.5% of the cases. Type x was found in 18.6%; supply by the STA in 8.5%, by a TIA in 1.9% and directly from the thyroid gland in 8.2% of the cases. If the blood supply was directly from the thyroid gland, parathyroid tissue and the blood vessels were proven by histological examination (Fig. 5).

56 laryngeal compounds have been from female body donors, 43 from male: Type a was present in 70% and 61.1%, type b in 14.4% and 16.7% and type x in 15.6% and 22.2%. Four compounds could not be assigned to a sex anymore.

### Anatomic mapping and surgical application

#### Anatomic mapping

**Figure 6:**
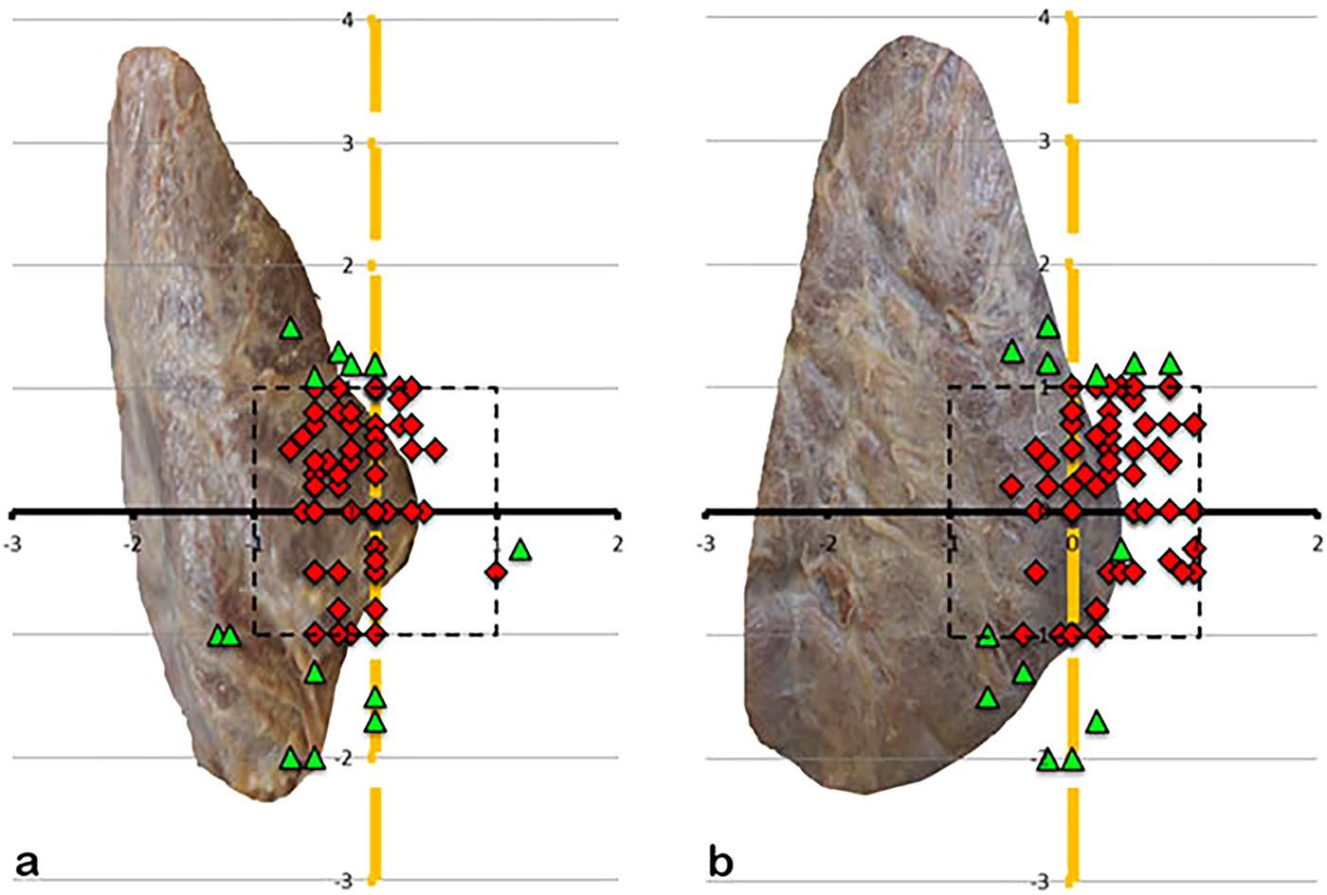
(**a**,**b)** Showing the position of the left SPG, (**a)** view from dorsal, (**b)** view from lateral, x-axis ZT-level, y-axis ILN-level, 1-cm scale. The red squares are showing the positioning of the superior parathyroids within 1 cm perimeter (dotted rectangle), the green triangles outside 1 cm perimeter.

**Figure 7:**
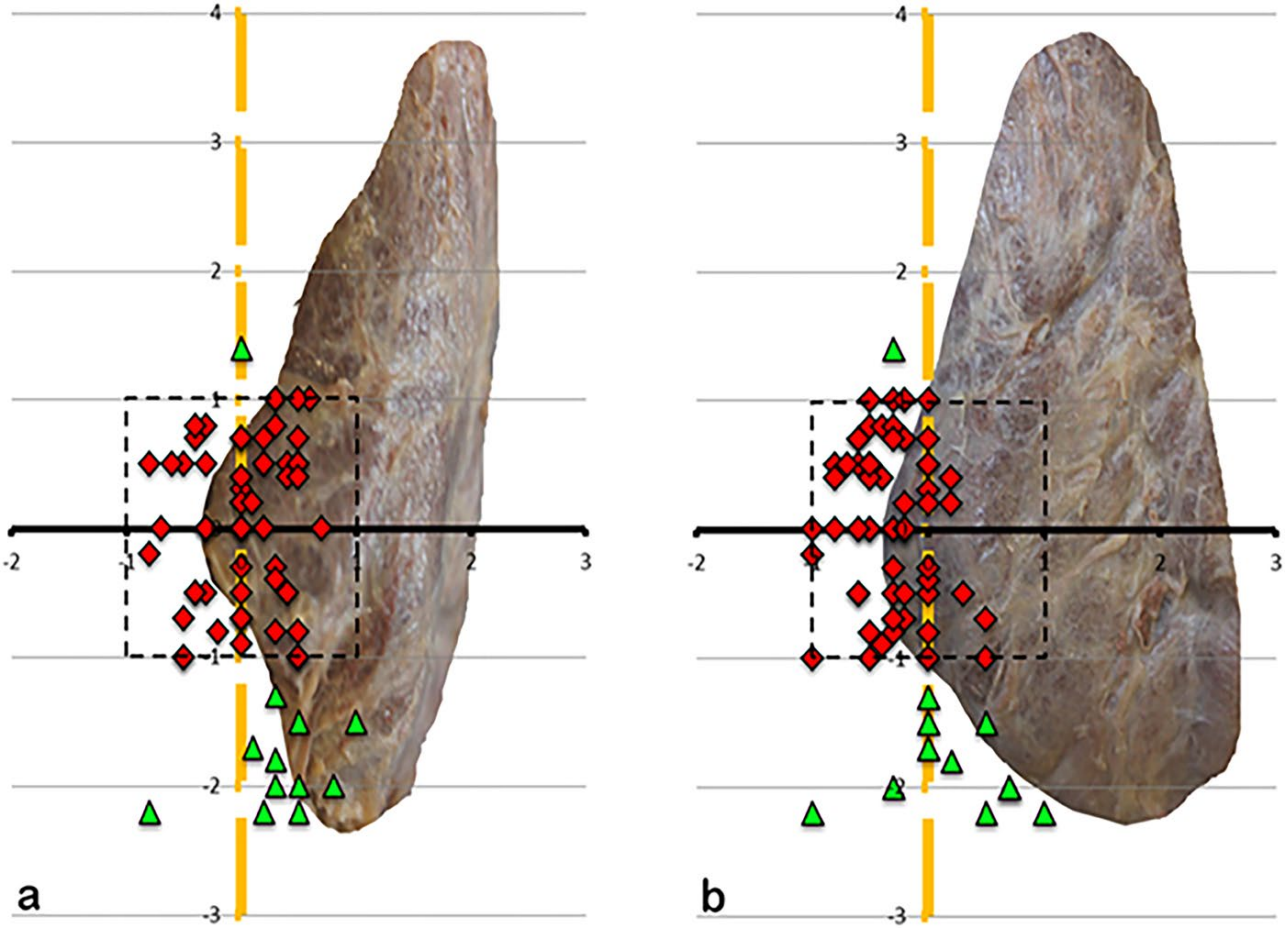
(**a**,**b)** Showing the position of the right SPG, (**a)** view from dorsal, (**b)** view from lateral.

**Figure 8:**
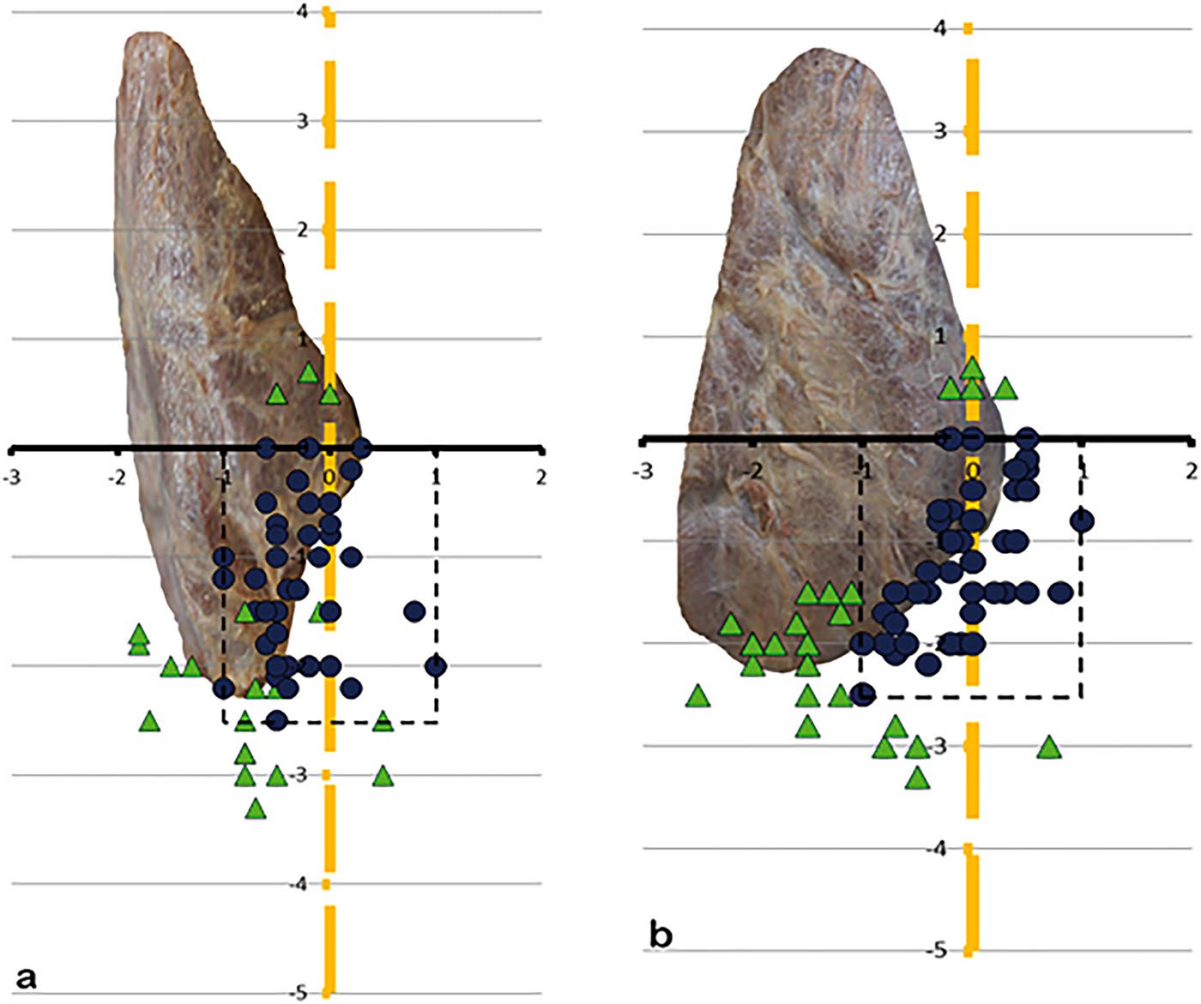
(**a**,**b)** Showing the position of the left IPG, (**a)** view from dorsal, (**b)** view from lateral, x-axis ZT-level, y-axis ILN-level, 1-cm scale. The blue dots are showing the positioning of the inferior parathyroids within 1 cm perimeter of the ILN-level and within 2,5 cm perimeter (dotted rectangle) on the ZT-level, the green triangles outside the perimeter.

**Figure 9:**
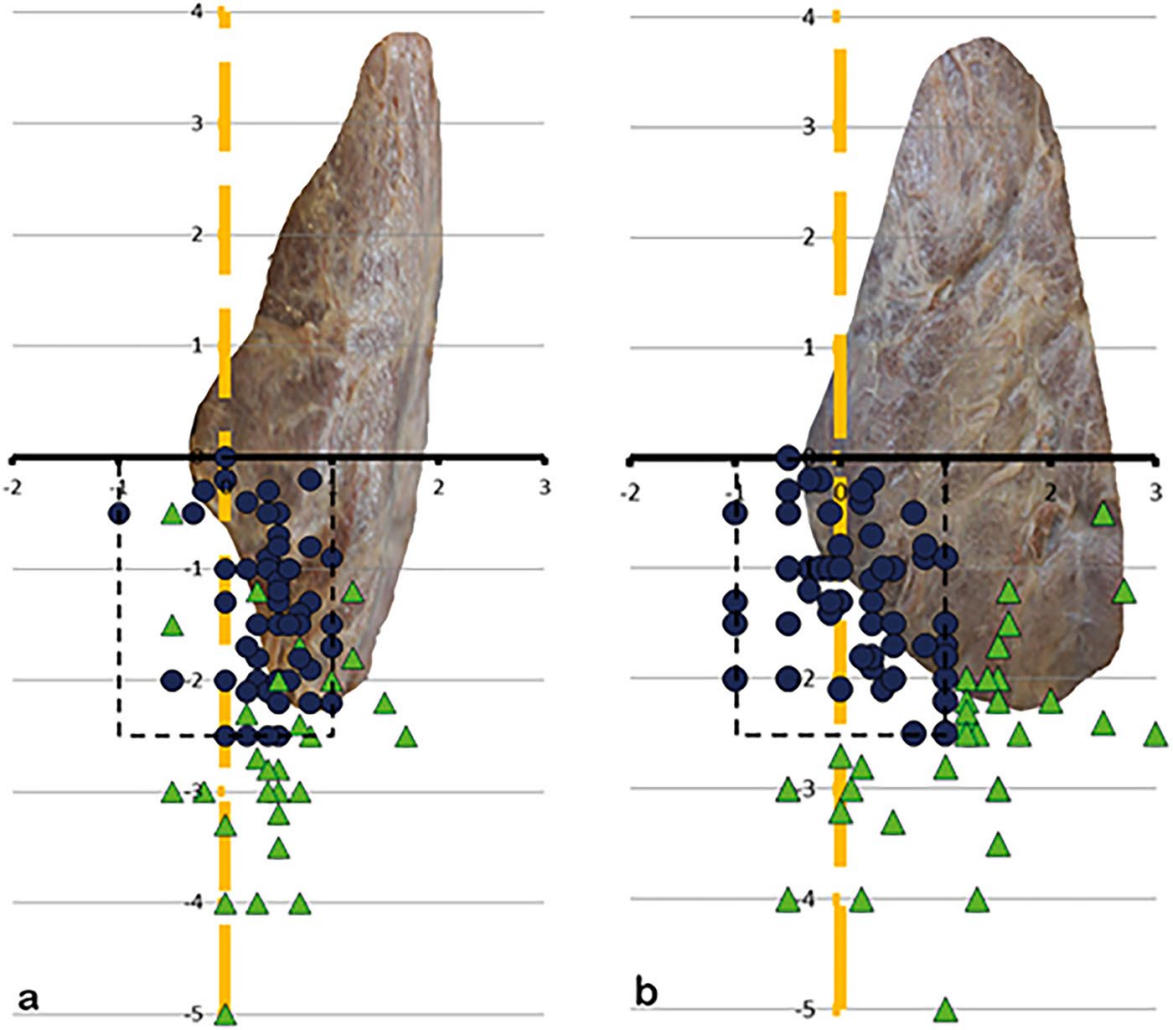
(**a**,**b**) Showing the position of the right IPG, (**a)** view from dorsal, (**b**) view from lateral.

81.8% of the left SPG (Fig. 6a,b) and 84.2% of the right SPG (Fig. 7a,b) lied within an 1 cm-perimeter of the ILN and 1 cm caudal and cranial of the ZT-level. So, during 3/4 of surgeries, there will be an 83% chance of finding the SPG inside the perimeter (95% CI, 0,76–0,89; p = 0,012). 65.8% of the left IPG (Fig. 8a,b) and 63.8% of the right IPG (Fig. 9a,b) lied within an 1 cm-perimeter of the ILN and on the ZT-level respectively 2.5 cm below, which means a greater variability.

These perimeters mark the crucial areas along the ILN during thyroid and parathyroid surgery (Table 2).

**Table 2:** Numbers of parathyroids lying close to the inferior laryngeal nerve and their blood supply.

	left SPG	left IPG	right SPG	right IPG
Proximity to ILN	63 (81.8%)	50 (65.8%)	64 (84.2%)	56 (63.6%)
Type of blood supply	22 a (35%)	38 a (76%)	41 a (64%)	46 a (82%)
	20 b (32%)	5 b (10%)	15 b (23%)	4 b (7%)
	21 × (33%)	7 × (14%)	8 × (13%)	6 × (11%)

**Figure 10:**
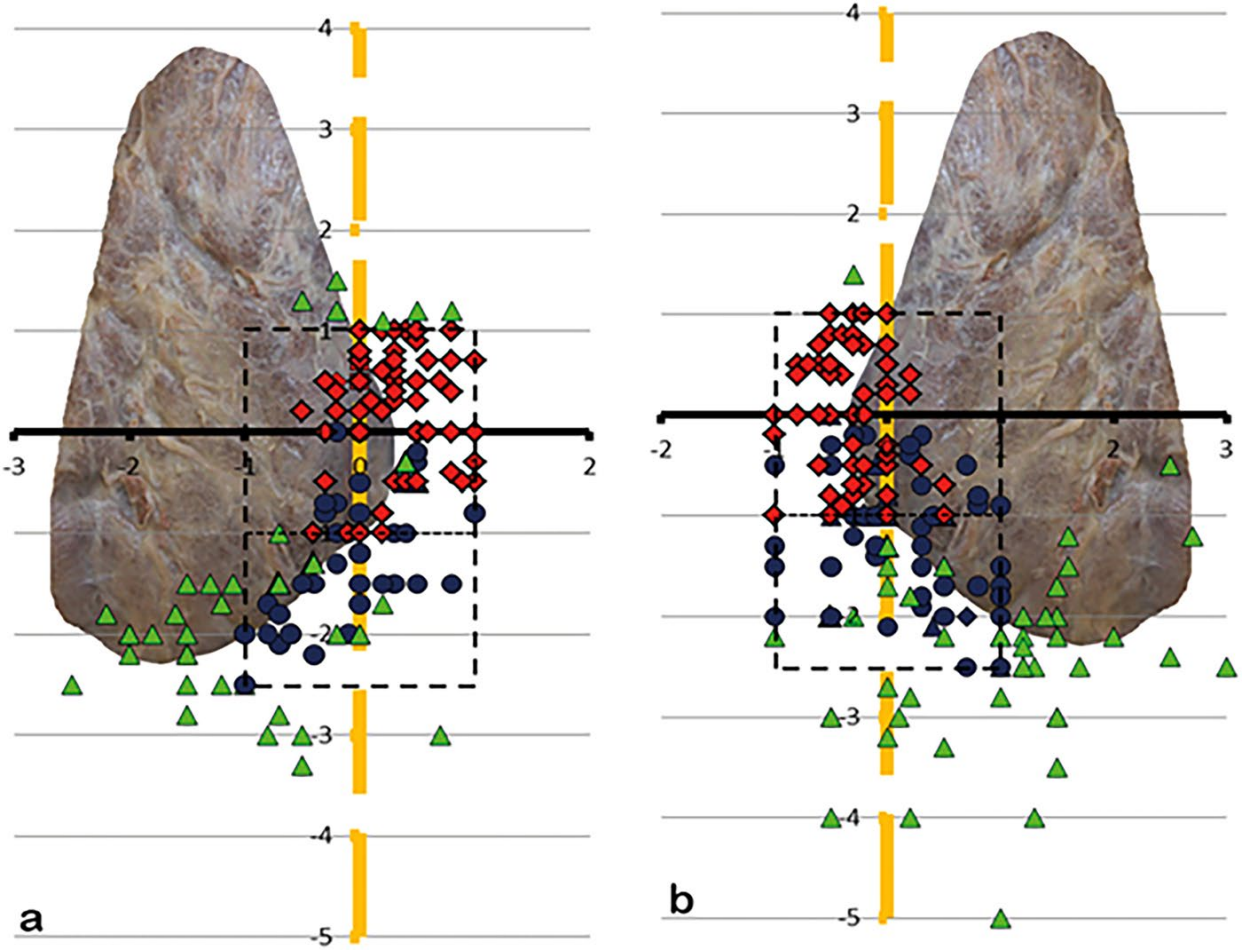
(**a**,**b)** Picture of the arrangement of the SPG and IPG on 10 a the left and 10 b the right side when seen from lateral (SPG – squares, are within the perimeter (dotted rectangle); IPG – dots, are inside the perimeter, triangles, regardless of being SPG or IPG, are outside).

83% of all superior parathyroids lied dorsal and 72.5% of all inferior parathyroids lied ventral of the ILN or on its level when seen from lateral (Fig. 10a,b).

35% of the left SPG and 64% of the right SPG lying within the upper perimeter as well as 76% of the left IPG and 82% of the right IPG lying within the lower perimeter receive their blood exclusively from the ITA. Anastomoses with the STA are present in 32% and 23% (upper perimeter) as well as 10% and 7% (lower perimeter) of the cases (Table 2).

**Figure 11:**
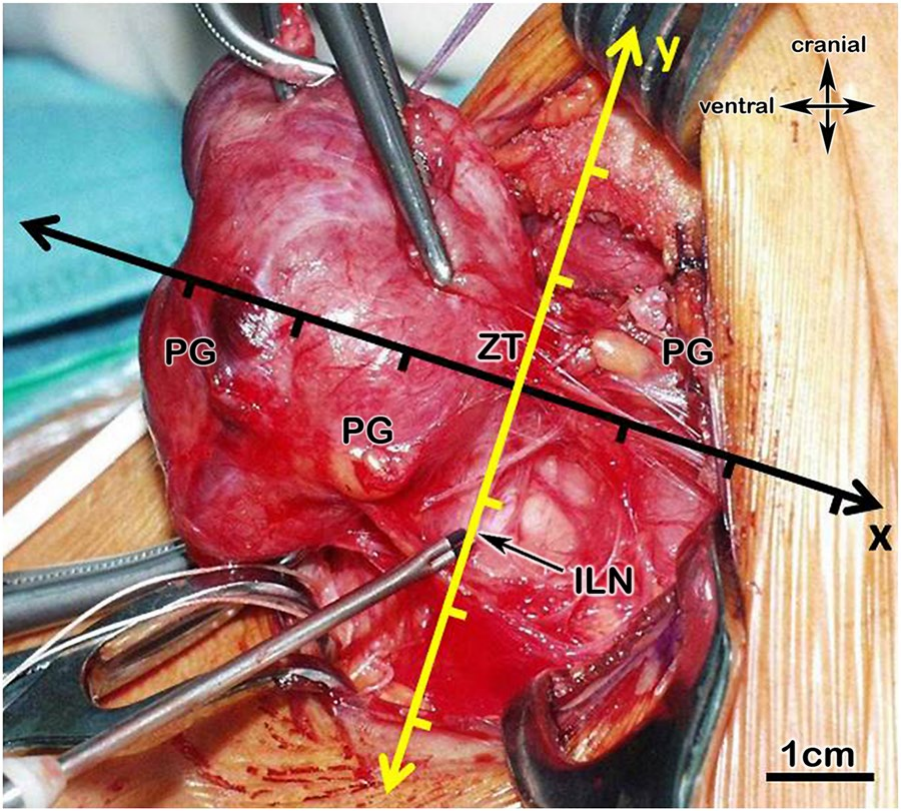
Intraoperative case, with the coordinate system attached, of a left thyroid lobe showing 3 parathyroids. The lower ones on the left. 1 parathyroid (dark coloured) lies intracapsular (histological proved) and is already changing its colour.

Beside our main objectives, we want to point out, that the amount of parathyroids is highly variable as we found supernumerary parathyroids in 8 cases (1 × 10, 1 × 9, 1 × 6 and 5 × 5) with 4 additional SPG and 14 IPG. Figure 11 shows an intraoperative case of a left thyroid lobe.

### Surgical application and “quick tip”

**Figure 12:**
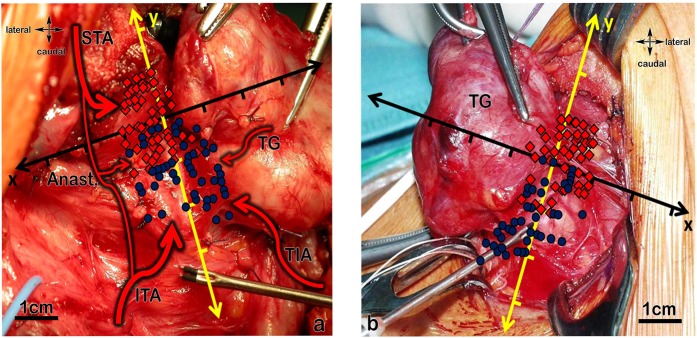
(**a**,**b)** Gives an overview and connects our results of the artery pattern and the location of the parathyroids to intraoperative field. (**a)** Overview of the location on the right side including blood supply: view from lateral on the right thyroid lobe, the red squares are showing the positioning of the superior parathyroids within 1 cm perimeter, the blue dots the positioning of the inferior parathyroids within 1 cm perimeter of the inferior laryngeal nerve-level (yellow-axis) and within 2,5 cm perimeter on the Zuckerkandl tubercle-level (black-axis); possible artery pattern of the parathyroids: blood supply by the inferior thyroid artery (ITA); blood supply by anastomoses between inferior thyroid artery and superior thyroid artery (Anast.); blood supply by the superior thyroid artery (STA); blood supply directly out of the thyroid gland (TG); blood supply by a thyroid ima artery (TIA). (**b)** Overview of the location on the left side (possible artery pattern same as on the right): view from lateral on the left thyroid lobe, the red squares are showing the positioning of the superior parathyroids within 1 cm perimeter, the blue dots the positioning of the inferior parathyroids within 1 cm perimeter of the inferior laryngeal nerve-level (yellow) and within 2,5 cm perimeter on the Zuckerkandl tubercle-level (black); TG…thyroid gland.

The most important clinical implication will be to not miss the parathyroid gland during thyreoidectomy or other surgical procedures in this region of the neck (Fig. 12a,b). A golden rule in endocrine surgery is to dissect without any bleeding, because if the operative field is monochromatic red it is not possible to differentiate between the relevant anatomic structures as parathyroid glands and recurrent laryngeal nerve. Thyroid surgery is first surgery of the parathyroids and recurrent laryngeal nerve. By starting thyroid surgery the “Grenzlamelle of Gemsenjäger”^18^ should be identified and dissected laterally, then the parathyroids can be identified and dissected away from the thyroid gland. It is not necessary to identify every parathyroid gland, because some of them are located ectopically, but no parathyroid gland should be overlooked. If one parathyroid cannot be protected in its blood supply it should be autotransplantated into the strap muscles or into the sternocleidoid muscle. Thereby it is very important to cut the parathyroid tissue in small pieces because initially nutrition is only by means of diffusion and diffusion reaches almost six cell layers. Every parathyroid gland should be regarded as being the last one, this means even if three glands are regarded as viable and the fourth is impaired in its blood supply this parathyroid has to be autotransplanted. Figure 12 gives an overview and connect our results of the artery pattern and the location of the parathyroids to intraoperative field.

## Discussion

Transient or permanent hypoparathyroidism remain one of the most common and important complications in thyroid surgery. Our study shows the importance of anatomic knowledge especially in the cervical regions during surgery for possible prevention of these complications providing a surgical mapping. Although in many studies the ZT wasn’t present in all cases^19^, we always took the most medial part of the posterior portion of the TG for measurement and defined it as a “ZT”^20^. This enables the surgeon to apply our Cartesian coordinate system during every surgery, even if a (big) “ZT” is not clearly visible.

Halsted *et al*. described the average position of the superior parathyroids at the level of the junction of the upper and middle thirds of the lateral lobe of the thyroid gland and the inferior ones between the middle of the lobe and its lower pole or even below^9^. Our research can confirm the results for the inferior parathyroids whereas the superior parathyroids mainly accumulate around the medial margin of the middle third. The inferior parathyroids underlie a larger variation, which makes them less exposed during surgery. The position of the superior parathyroid gland is usually dorsal of the thyroid gland, below the upper pole at the level of the cricoid cartilage. The localisation of the inferior parathyroid gland is more fluctuating but mostly dorsal and close to the lower pole of the thyroid gland or along the thyreothymic ligament^4^. Nevertheless, about 16% of the parathyroids may lay ectopic^21^. Intraoperative techniques like staining the parathyroids with methylene blue^22^ or using near-infrared imaging for identification^23^ are facilities to support the surgeon, but can never replace her or his anatomic knowledge.

Yun *et al*. also took the ZT as a main landmark on which the position of the superior parathyroids is referring to and the tracheoesophageal groove as a y-axis. They found the most superior parathyroids lying between 1 and 3 o’clock on the left and 10 and 11 o’clock on the right side^19^ which means, that they mainly lay dorsal of the ZT. This matches with our results although we defined the ILN as y-axis due to its more constant anatomic relation to the ZT. Furthermore, the ILN is a more important structure during surgery than the tracheoesophageal groove and therefore, the surgeon will give more attention to the nerve.

Within the mentioned perimeters, the ITA provides blood in most cases, what makes a preoperative identification of the artery with ultrasonography advisable.

However, the detection of normal parathyroids with ultrasonography is disputable, so the mentioned coordinate system seems to be a useful and simple way to make intraoperative identification easier and lower the risk of hypoparathyroidism.

A reason for the adjacency of the superior parathyroids to the ZT, a remnant of the ultimobranchial body, could be their shared origin from the 4^th^ branchial cleft^24^, whereas the inferior descents from a longer way from the 3^rd^ branchial cleft and therefore show larger variation^25^.

As mentioned in our results, 33.8% and 10.5% of thesuperior parathyroids received their blood from another vessel. But why is there an imbalance between left and right? In 9 cases the left ITA was absent and therefore, the left STA had to take over the blood supply completely.

The venous drainage of the parathyroids is always neglected although a study by Lee *et al*. showed that the preservation of the inferior thyroid veins during surgery lowers the risk of postoperative hypocalcaemia and furthermore, hypocalcaemic patients reach a normal calcium level quicker after surgery^26^.

Cui *et al*. in their prospective cohort study described the blood supply of the parathyroids as well, setting their focus on whether the supply is dependent on the thyroid gland or not^15^. So, they defined 5 types, from Type A with no dependency on the thyroid over B1–3 with partial to mostly blood supply from the thyroid to C, where the parathyroids receive their blood completely from the thyroid. Depending on the type of blood supply, the parathyroids can be kept *in-situ* or must be autografted. Their findings, that 6.61% of the parathyroids receive their blood solely from the thyroid are correlating with our results (8.2%)^15^.

In case of goitered thyroids, the inferior parathyroids can diverge to the lateral side of the lobes, respectively caudal of the inferior pole whereas the superior parathyroids mainly stay medial but can move towards the inferior pole as well^27^.

In quite a few cases it was difficult to differ between parathyroids and accessory thyroid glands. If we were in doubt, we made a histologic examination for proof of parathyroid tissue. They mainly lie close to the medial margin of the thyroids and aren’t connected via glandular tissue but covered with an own fascia^28^.

Of course it has to be pointed out that the incidence of postoperative hypocalcemia and even hypoparathyroidism is also depending on the difficulty of the surgical case, the experience and ability of the surgeon him or herself to translate his or her implicit knowledge into lower complication rates^29^. These risks, like sex, younger age, autografting of 1 or 2 parathyroids, central lymph node dissection, can also be predicted during thyroid surgery, which was evaluated by Pomberger *et al*.^29^. They could show that surgeons assess the risk and adapt their surgical “intraoperative decision-making process” accordingly. Nevertheless, a good-understood anatomic knowledge of the blood supply of the parathyroid glands and their topographic relationships to the thyroid gland might be the indispensable prerequisite for every endocrine surgeon operating in the neck to avoid postoperative complications like hypoparathyroidism.

The biggest limitation of the study is the fact, that the authors didn’t look for ectopic or intrathyroidal parathyroids. On the other hand, this has no impact on the results, as the goal was to focus on the situs for thyroid surgery only.

## Conclusions

Concerning the blood supply of the parathyroids, the ITA is the main supplying artery although in many cases anastomoses from the STA provide a “backup”-supply at least for the superior parathyroids. Some parathyroids receive their blood supply directly from the thyroid gland (8.2%), which means that they can’t be preserved during thyroidectomy. If identified, they need to be autografted, which might avoid transient or permanent postoperative hypoparathyroidism. Our anatomic mapping enables the surgeon to apply it during surgery and gives a better orientation for the localisation of the parathyroids, in order to protect them.

## Material and Methods

The bodies were donated by individuals to the Division of Clinical and Functional Anatomy of the Medical University of Innsbruck who had given their written informed consent prior to death for their use for scientific and educational purposes^30,31^. All cadavers were preserved using an arterial injection of a formaldehyde solution and immersion in phenolic acid in water for one to three months. The possibility of this solution causing preservation artefacts can be denied^32^. The bodies are a representative sample of the general Austrian and German population at the age of death^30^. According to Austrian National Law, scientific institutions (in general Institutes, Departments or Divisions of Medical Universities) are entitled to receive the body after death mainly by means of a specific legacy, which is a special form of last will and testament. No bequests are accepted without the donor having registered their legacy and been given appropriate information upon which to make a decision based upon written informed consent (policy of ethics)^31^; therefore an ethics committee approval is not necessary.

### Blood supply

103 formalin-phenol fixed laryngeal compounds have been examined in order to classify the type of blood supply of the parathyroids and their topographic location compared to the inferior laryngeal nerve (ILN) and the Zuckerkandl tubercle (ZT). Ectopic parathyroids, as in the thorax, inside the carotid sheath or along the thyreothymic ligament have not been examined due to the fact that the main focus lay on the structures directly affecting thyroid surgery only. Intrathyroidal parathyroids have not been examined as well because they can’t be preserved during surgery. If in doubt, we slit the specimen to identify parathyroid tissue or made histological examinations for verification. Goitered thyroid glands have only been examined when the ZT was clearly visible. Examination bias has been avoided due to the fact, that the same experienced person has dissected all anatomical laryngeal compounds. Surgical magnifying glasses, microscopes, tweezers, scissors and scalpels have been used.

The dissections always followed the same scheme: After identification of all arterial vessels and assessment of whether the dissection is suitable, the search for parathyroids was started. The fasciae and fat were carefully removed from dorsomedial to lateral, in order to protect the vessels and to get in between the thyroid and the larynx. Thereafter, the arteries were dissected from proximal to distal following all small vessels until the parathyroids were found. If absent, the organ capsule of the thyroid gland was opened and searching for the parathyroids continued.

### Anatomic mapping

If we found a single parathyroid on one side, we named it a “superior” if above the ZT-level, and “inferior” if below. In the case of two parathyroids on one side, the upper one was the “superior” and the lower one the “inferior”, even if it was above the ZT-level and vice versa. From a pure embryological point of view it seems not appropriate but reasonable for the application during surgery, that the upper parathyroid may be the evolutionary lower one. For the mapping, the ZT-plane forms the x-axis whereas the inferior laryngeal nerve forms the y-axis. In order to make data more understandable, the nerve must be seen as a vertical and elongated line, even above its bend into the larynx. Thus, this two structures form a Cartesian coordinate system, which makes intraoperative orientation easier. (Figs 1a,b and 2).

Statistics have been calculated with Clopper-Pearson interval and one-tailed binomial test.

### Ethical approval

All procedures performed in studies involving human participants were in accordance with the ethical standards of the institutional and/or national research committee and with the 1964 Helsinki declaration and its later amendments or comparable ethical standards.

### Informed consent

Informed consent was obtained from all individual participants included in the study.

According to Austrian National Law, scientific institutions (in general Institutes, Departments or Divisions of Medical Universities) are entitled to receive the body after death mainly by means of a specific legacy, which is a special form of last will and testament. No bequests are accepted without the donor having registered their legacy and been given appropriate information upon which to make a decision based upon written informed consent (policy of ethics); therefore an ethics committee approval is not necessary.

## Supplementary information

Tables 1+2

## Data Availability

The datasets generated during and/or analysed during the current study are available from the corresponding author on reasonable request.
