# Supplementary material for: Postoperative Hypoparathyroidism in Thyroid Surgery: Anatomic-Surgical Mapping of the Parathyroids and Implications for Thyroid Surgery
Source: Sci Rep. 2019 Oct 30;9:15700. doi: 10.1038/s41598-019-52189-3 (PMC6821921; doi:10.1038/s41598-019-52189-3)
Supplement: Supplementary file 1 — Tables 1+2 [file 41598_2019_52189_MOESM1_ESM.docx]

# Tables

|  | **Amount** | **Type a** | **Type b** | **Type x** | ***TG*** | ***STA*** | ***TIA*** |
| --- | --- | --- | --- | --- | --- | --- | --- |
| **left SPG** | 77 | 40.2 % | 26 % | 33.8 % | *14.3 %* | *18.2 %* | *1.3 %* |
| **left IPG** | 76 | 72.4 % | 7.9 % | 19.7 % | *4 %* | *11.8 %* | *3.9 %* |
| **right SPG** | 76 | 65.8 % | 23.7 % | 10.5 % | *7.9 %* | *2.6 %* | *-* |
| **right IPG** | 88 | 82.9 % | 5.7 % | 11.4 % | *6.8 %* | *2.3 %* | *2.3 %* |

**Table 1**: Types of blood supply

**Table 2:** Numbers of parathyroids lying close to the inferior laryngeal nerve and their blood supply

|  | **left SPG** | **left IPG** | **right SPG** | **right IPG** |
| --- | --- | --- | --- | --- |
| **Proximity to ILN** | 63 (81.8 %) | 50 (65.8 %) | 64 (84.2 %) | 56 (63.6 %) |
| **Type of blood supply** | 22 a (35 %) | 38 a (76 %) | 41 a (64 %) | 46 a (82 %) |
|  | 20 b (32 %) | 5 b (10 %) | 15 b (23 %) | 4 b (7 %) |
|  | 21 x (33 %) | 7 x (14 %) | 8 x (13 %) | 6 x (11 %) |
